# Degradation of Fucoxanthin to Elucidate the Relationship between the Fucoxanthin Molecular Structure and Its Antiproliferative Effect on Caco-2 Cells

**DOI:** 10.3390/md16080275

**Published:** 2018-08-06

**Authors:** Shiro Komba, Eiichi Kotake-Nara, Wakako Tsuzuki

**Affiliations:** Food Component Analysis Unit, Food Research Institute, National Agriculture and Food Research Organization, 2-1-12, Kannondai, Tsukuba, Ibaraki 305-8642, Japan; ekotake@affrc.go.jp (E.K.-N.); wakako@affrc.go.jp (W.T.)

**Keywords:** fucoxanthin, ozonolysis, apo-fucoxanthinone, Caco-2, antiproliferative effect

## Abstract

Fucoxanthin has an antiproliferative effect on cancer cells, but its detailed structure–activity correlation has not yet been elucidated. To elucidate this correlation, fucoxanthin was degraded by ozonolysis. The degraded compounds of fucoxanthin obtained by ozonolysis were purified by HPLC and analyzed by NMR. The polyene chain of fucoxanthin was cleaved by ozonolysis, and the fucoxanthin was divided into two types of cyclohexyl derivatives, one with a β,γ-epoxy ketone group and the other with an allenic bond. In order to elucidate the structure–activity correlation, Caco-2 cells (human colorectal carcinoma) were treated with fucoxanthin degradation compounds. It was found that the entire structure of fucoxanthin is not essential for its antiproliferative effect and that even a partial structure exerts this effect.

## 1. Introduction

Fucoxanthin (**1**) is a xanthophyll-type carotenoid which is mainly distributed in brown algae [1,2,3,4,5,6,7,8,9,10]. It has been reported that it has anticarcinogenic [11,12,13,14,15,16,17,18,19,20,21,22], anti-obesity [23,24,25], anti-inflammatory [26], anti-angiogenic [27], and antioxidative [28] effects. We focused on the anticarcinogenic activity of fucoxanthin.

It is well known that fucoxanthin and its metabolite, fucoxanthinol, induce G_1_ cell-cycle arrest and apoptosis in various cell lines and can inhibit cancer development in animal models [22]. However, its detailed structure–activity correlation has not been elucidated, particularly regarding what kind of molecular structure of fucoxanthin is responsible for this activity. Therefore, we decided to decompose a fucoxanthin molecule in order to elucidate the detailed structure–activity relationship and elucidate the mechanisms involved in its antiproliferative effect. By investigating the antiproliferative effect using each part of the degraded fucoxanthin, the structure contributing to the activity was able to be elucidated.

Fucoxanthin has a very complicated structure, with an allenic bond, a polyene chain, an acetyl, and a β,γ-epoxy ketone group. In addition, the two six-membered ring derivatives bound by the polyene chain are not symmetrical, with one having an allenic bond and the other having a β,γ-epoxy ketone group. In particular, we focused on the two six-membered ring derivatives that are connected by a polyene chain. We anticipated that one of the two might contribute to this activity and separated them by cutting the polyene chain. In regard to its anti-obesity activity, the allenic bond on fucoxanthin may be a key structure, according to Miyashita et al. [25]. Using these two six-membered ring derivatives, the antiproliferative effect on Caco-2 cells was examined. Although Caco-2 cells have been widely used as a model of intestinal absorption, we used them here as colon cancer cells, as we did in our previous study [29].

In order to chemically decompose fucoxanthin, which is sensitive to light, acid, and basic conditions, it was necessary to perform the reaction under mild, neutral conditions. Therefore, ozone oxidation was chosen, in which the reaction proceeds in a neutral condition at a relatively low temperature, and only gas is used as a reagent. Ozone oxidation is a reaction involving the oxidization of a double bond to decompose it into two aldehydes. It was predicted that fucoxanthin has a polyene chain, and that ozone easily oxidizes the double bond and decomposes it into two six-membered ring derivatives.

## 2. Results and Discussion

### 2.1. Chemistry

**Figure 1:**
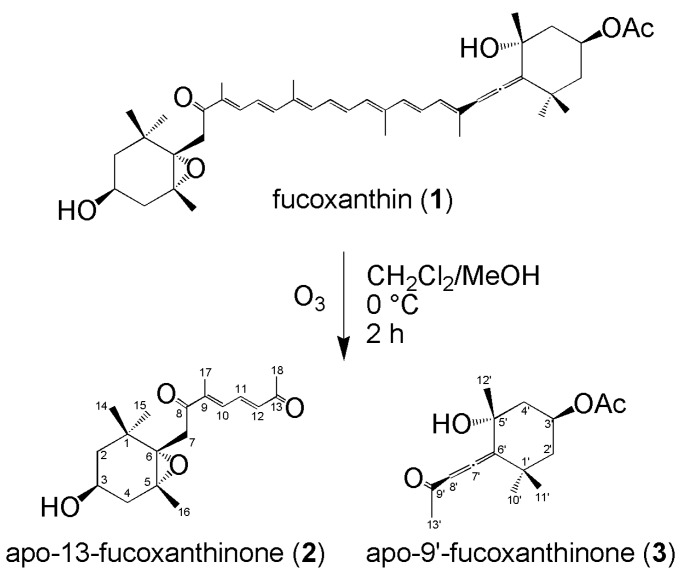
Synthesis of two six-membered ring derivatives (apo-13-fucoxanhinone (**2**) and apo-9′-fucoxanthinone (**3**)).

**Figure 2:**
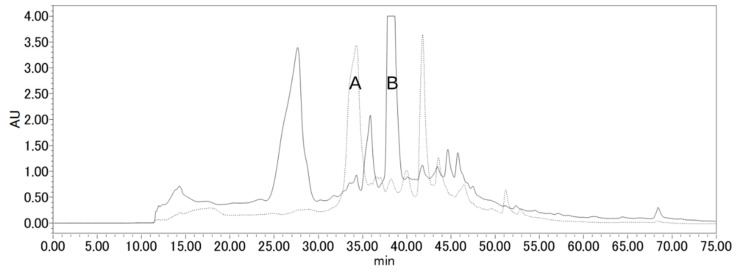
HPLC profile of fucoxanthin degradation compounds. Column: Mightysil RP-18 GP 250-20 column (20 × 250 mm, 5 μm; Kanto Chemical, Tokyo, Japan); solvent A: water; solvent B: EtOH. The solvent profile was as follows (linear gradient between each step): initial conditions: A/B = 75:25, 1 mL/min; 1 min: A/B = 75:25, 5 mL/min; 10 min: A/B = 75:25, 5 mL/min; 60 min: A/B = 0:100, 5 mL/min. A straight line indicates an absorbance of 215 nm. The dashed line indicates an absorbance of 280 nm. Peak A: apo-13-fucoxanthinone (**2**) (34.3 min); peak B: apo-9′-fucoxanthinone (**3**) (38.6 min).

Fucoxanthin was purified from wakame (*Undaria pinnatifida*) (1.09 mg fucoxanthin/g dry wakame), as referenced in previous experiments [30]. The ^1^H-NMR and ^13^C-NMR spectra were identical to those previously reported. Purified fucoxanthin was dissolved in dichloromethane/methanol, and ozone gas was bubbled through the stirred mixture at 0 °C for 2 h (Figure 1). We then replaced ozone gas with nitrogen gas, and dimethyl sulfide was added as a reducing agent. Silica gel was added to the reaction mixture to separate the polar substances. The degradation products of fucoxanthin were purified by reverse phase HPLC by detecting the absorbance at both 215 nm and 280 nm (Figure 2). Though many peaks appeared on the HPLC chart, the main peaks were collected and analyzed by NMR. Peak A in Figure 2, which has a strong absorbance at 280 nm, was obtained at 1.87 mg (6.9% yield). On the other hand, peak B in Figure 2, which has a strong absorbance at 215 nm, was obtained at 7.85 mg (33% yield). From the ESI-Orbitrap-MS analysis and ^1^H-NMR, COSY-NMR, ^13^C-NMR, HSQC-NMR, and HMBC-NMR analyses, peak A in Figure 2 was identified as apo-13-fucoxanthinone (**2**) [4] (Table 1) and peak B in Figure 2 was identified as apo-9′-fucoxanthinone (**3**) [4] (Table 2). The NMR spectrum of apo-13-fucoxanthinone (**2**) (Figure S1), which has five methyl groups {*δ* = 0.94 (s, 3H, Me-15), 1.03 (s, 3H, Me-14), 1.21 (s, 3H, Me-16), 2.03 (s, 3H, Me-17), 2.35 (s, 3H, Me-18) ppm}, three methylene groups {*δ* = 1.34 and 1.50 (each m, 2H, H-2a and H-2b), 1.79 and 2.31 (each m, 2H, H-4a and H-4b), 2.61 (d, 1H, *J*_gem_ 18.6 Hz, H-7a), 3.63 (d, 1H, *J*_gem_ 19.0 Hz, H-7b) ppm}, three methine groups {*δ* = 6.46 (d, 1H, *J*_11,12_ 15.4 Hz, H-12), 7.05 (d, 1H, *J*_10,11_ 11.2 Hz, H-10), 7.47 (dd, 1H, *J*_10,11_ 11.3 Hz, *J*_11,12_ 15.4 Hz, H-11) ppm} and one oxymethine group {*δ* = 3.81 (m, 1H, H-3) ppm}, agreed well with the NMR data of the compound reported by Mori et al. [4]. In addition, the NMR spectrum of apo-9′-fucoxanthinone (**3**) (Figure S2), which has four methyl groups {*δ* = 1.16 (s, 3H, Me-10′), 1.43 (s, 6H, Me-11′ and Me-12′), 2.19 (s, 3H, Me-13′) ppm}, one acetyl group {*δ* = 2.05 (s, 3H, Ac) ppm}, two methylene groups {*δ* = 1.44 (1H, H-2′a), 1.53 (dd, 1H, *J* 11.4 Hz, *J* 12.9 Hz, H-4′a), 2.02 (dd, 1H, *J* 2.2 Hz, *J* 4.3 Hz, H-2′b), 2.33 (ddd, 1H, *J* 2.2 Hz, *J* 4.3 Hz, *J* 12.9 Hz, H-4′b) ppm}, one methine group {*δ* = 5.87 (s, 1H, H-8) ppm}, and one oxymethine group {*δ* = 5.39 (tt, 1H, *J* 4.2 Hz, *J* 11.5 Hz, H-3′) ppm} also agreed well with the NMR data of the compound reported by Mori et al. [4]. Since these two types of degradation product have already been found in edible brown algae cultivated in deep seawater by Mori et al. [4], there is a possibility that this experiment artificially reproduced the decomposition of fucoxanthin which occurs in nature. Furthermore, based on the discovery made by Mori et al. and the results from this experiment, positions 13 and 9′ of fucoxanthin are easily oxidatively cleavable sites in the polyene chain of fucoxanthin. As a result, we succeeded in separating the two six-membered rings.

### 2.2. Biology

**Figure 3:**
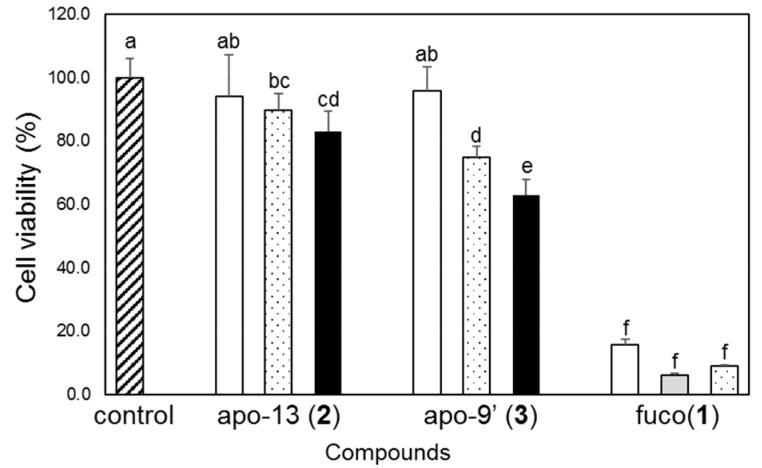
Concentration-dependent antiproliferative effect of apo-13-fucoxanthinone (apo-13, **2**), apo-9′-fucoxanthinone (apo-9′, **3**), and fucoxanthin (fuco, **1**) on Caco-2 cells. Caco-2 cells were cultured with apo-13 (**2**), apo-9′ (**3**) and fuco (**1**) in supplemented medium for 72 h. Cell viability was estimated by MTT assay and is expressed relative to the control cells treated with the vehicle alone. The pattern of each bar shows the concentration, as follows: 0 μM: hatched bar, 10 μM: open bar, 20 μM: shaded bar, 30 μM: dotted bar, 50 μM: solid bar. The data represent the mean ± standard deviation of three wells. Replicate experiments demonstrated similar trends. Values not sharing common alphabets (from a to f) were shown to be significantly different with the Tukey–Kramer test (*p* < 0.05).

These obtained compounds were evaluated for their antiproliferative activity using Caco-2 cells. After 72 h of cultivation, cell viability was evaluated by 3-(4,5-dimethylthiazol-2-yl)-2,5-diphenyl tetrazolium bromide (MTT) assay [31] (Figure 3). As a result, it was found that each of the degraded compounds inhibited the proliferation of Caco-2 cells in a concentration-dependent manner. In addition, apo-9′-fucoxanthinone (**3**) was found to inhibit proliferation more strongly than apo-13-fucoxanthinone (**2**). However, its activity was weaker than that of the original fucoxanthin (**1**). Both structures may be necessary to exert a powerful effect like that of fucoxanthin. The synergistic effect of these two degraded compounds will be discussed in the future. Although the activity of apo-13-fucoxanthinone (**2**) has not been studied extensively, the activity of apo-9′-fucoxanthinone (**3**) has recently drawn attention. For example, anti-inflammatory effects [32,33,34] and hair growth effects [35] have been reported. Detailed examination of the fucoxanthin when decomposed will lead to the discovery of new activity of the degradation products. Furthermore, this study suggests that not only fucoxanthin itself, but also the degradation products, may exert effects as a mechanism of fucoxanthin’s activity in humans.

## 3. Materials and Methods

### 3.1. Reagents and Conditions

^1^H NMR and ^13^C NMR spectra were obtained in CDCl_3_ on a Bruker BioSpin spectrometer (AV 400, Bruker Corporation, Madison, MA, USA). Chemical shifts are given in ppm and referenced to Me_4_Si (δ 0.00). The following abbreviations are used for the characterization of NMR signals: s = singlet, d = doublet, t = triplet, m = multiplet. The ozone generator was made by combining the ozone-generating electrode ZC-60-MM (Silver Seiko Ltd., Tokyo, Japan) with a non-noise s500 air pump (approximately 1 L/min) (Japan Pet Design Co., Ltd., Tokyo, Japan). ESI-Orbitrap-MS spectra were recorded on a Thermo Fisher Scientific instrument (VELOS PRO, Thermo Fisher Scientific Inc., Waltham, MA, USA). The optical rotations were determined in chloroform on a Jasco instrument (P-1020-GT, JASCO Corporation, Tokyo, Japan) under ambient temperature. Reverse-phase HPLC separation of degraded fucoxanthin compounds was performed using a Waters HPLC system with a Mightysil RP-18 GP 250-20 column (20 × 250 mm, 5 μm; Kanto Chemical Co., Inc., Tokyo, Japan) and detected at 215 nm and 280 nm, simultaneously. For the HPLC conditions, solvent A was water and solvent B was ethanol. Initially, 75% of solvent A at a flow rate of 1 mL/min was used; subsequently, the flow rate was increased to 5 mL/min for 1 min, and this condition was maintained for 10 min. Then, a linear gradient of 25% to 100% of solvent B was applied for 60 min, and this condition was maintained. The HPLC analysis of compounds in medium for the antiproliferation assay was performed as follows: fucoxanthin (**1**), apo-13-fucoxanthinone (**2**), and apo-9′-fucoxanthinone (**3**) were analyzed by HPLC (using an LC-20AT pump, an SPD-M10A photodiode array detector, and a CTO-10AS column oven at a constant temperature of 25 °C; Shimadzu Corporation, Kyoto, Japan) on an ODS-80Ts column (2.0 × 150 mm) with an ODS-S1 precolumn (2.0 × 10 mm; Tosoh Corporation, Tokyo, Japan). An isocratic analysis was performed at a flow rate of 0.2 mL/min with acetonitrile/methanol/water (75:15:10, *v*/*v*/*v*) containing 0.1% ammonium acetate for fucoxanthin (**1**) or acetonitrile/methanol/water (45:9:46, *v*/*v*/*v*) containing 0.1% ammonium acetate for apo-13-fucoxanthinone (**2**) and apo-9′-fucoxanthinone (**3**), respectively. These compounds were quantified from the peak area at 450 nm for fucoxanthin (**1**), 288 nm for apo-13-fucoxanthinone (**2**), and 232 nm for apo-9′-fucoxanthinone (**3**), respectively, using an authentic standard calibration curve. Dry wakame from China was purchased from a local market in Tsukuba, Japan. Human colorectal carcinoma Caco-2 cells were obtained from the American Type Culture Collection (Rockville, MD, USA). Dulbecco’s modified Eagle’s medium (DMEM) was purchased from Nissui Pharmaceutical Co., Ltd. (Tokyo, Japan). The DMEM used a low glucose-type (1.0 g/L glucose), so we increased the glucose concentration to 4.5 g/L. Tetrahydrofuran (THF) was purchased from Nacalai Tesque, Inc. (Kyoto, Japan). THF was purified in a neutral alumina column just before use. All reagents and solvents used were reagent grade.

### 3.2. Synthesis of Two Six-Membered Ring Derivatives {apo-13-Fucoxanthinone (***2***) and apo-9′-Fucoxanthinone (***3***)}

Fucoxanthin was purified from wakame (*Undaria pinnatifida*). The extraction solvents and purification method have been referenced in previous experiments [30]. A mixed solvent (chloroform/methanol/water 5:4:1) was used for the extraction and subsequent purification of the residue on the silica gel (ethyl acetate/toluene 1:5) through chromatography, resulting in pure fucoxanthin (1.09 mg/g dry wakame). The ^1^H-NMR and ^13^C-NMR spectra were identical to those previously reported. The purified fucoxanthin (58 mg, 88 μmol) was dissolved in dichloromethane/methanol (39 mL/66 mL) and cooled to 0 °C. Ozone gas from a homemade ozone generator was bubbled though the cooled and stirred mixture for 2 h. Afterwards, the ozone gas was replaced with nitrogen gas, dimethyl sulfide (130 μL, 1.77 mmol) was added to the mixture, and the mixture was stirred from 0 °C to room temperature for a further 2 h. Then, silica gel (35 g) was added to the mixture, and the solvent was removed under reduced pressure to absorb the reaction product into the silica gel. Thereafter, the polar substances were removed by silica gel column chromatography (ethyl acetate/*n*-hexane 2:1). The obtained syrup was purified by reverse phase HPLC to obtain compound **2** (apo-13-fucoxanthinone, 1.87 mg, 6.9%) and compound **3** (apo-9′-fucoxanthinone, 7.85 mg, 33%). All experiments were done under dim light in order to minimize the isomerization and degradation of fucoxanthin derivatives due to light irradiation. Compound **2** (apo-13-fucoxanthinone): ${\left[\alpha \right]}_{D}^{25}$ = −13 (*c* = 0.06, chloroform); ^1^H-NMR (400 MHz, CDCl_3_), *δ* = 0.94 (s, 3H, Me-15), 1.03 (s, 3H, Me-14), 1.21 (s, 3H, Me-16), 1.34 and 1.50 (each m, 2H, H-2a and H-2b), 1.79 and 2.31 (each m, 2H, H-4a and H-4b), 2.03 (s, 3H, Me-17), 2.35 (s, 3H, Me-18), 2.61 (d, 1H, *J*_gem_ 18.6 Hz, H-7a), 3.63 (d, 1H, *J*_gem_ 19.0 Hz, H-7b), 3.81 (m, 1H, H-3), 6.46 (d, 1H, *J*_11,12_ 15.4 Hz, H-12), 7.05 (d, 1H, *J*_10,11_ 11.2 Hz, H-10), 7.47 (dd, 1H, *J*_10,11_ 11.3 Hz, *J*_11,12_ 15.4 Hz, H-11); ^13^C-NMR (100 MHz, CDCl_3_), *δ* = 12.5 (C-17), 21.0 (C-16), 24.9 (C-14), 28.1 (C-15), 28.2 (C-18), 35.1 (C-1), 41.4 (C-7), 41.6 (C-4), 47.2 (C-2), 64.2 (C-3), 66.1 (C-5), 66.7 (C-6), 134.5 (C-10), 135.6 (C-12), 136.7 (C-11), 143.1 (C-9), 197.7 (C-13), 198.1 (C-8); ESI-Orbitrap-MS, calcd. for C_18_H_27_O_4_^+^ [M + H]^+^: 307.1904, found *m*/*z*: 307.1905. Compound **3** (apo-9′-fucoxanthinone): ${\left[\alpha \right]}_{D}^{25}$ = −19 (*c* = 0.08, chloroform); ^1^H-NMR (400 MHz, CDCl_3_), *δ* = 1.16 (s, 3H, Me-10′), 1.43 (s, 6H, Me-11′ and Me-12′), 1.44 (1H, H-2′a), 1.53 (dd, 1H, *J* 11.4 Hz, *J* 12.9 Hz, H-4′a), 2.02 (dd, 1H, *J* 2.2 Hz, *J* 4.3 Hz, H-2′b), 2.05 (s, 3H, Ac), 2.19 (s, 3H, Me-13′), 2.33 (ddd, 1H, *J* 2.2 Hz, *J* 4.3 Hz, *J* 12.9 Hz, H-4′b), 5.39 (tt, 1H, *J* 4.2 Hz, *J* 11.5 Hz, H-3′), 5.87 (s, 1H, H-8); ^13^C-NMR (100 MHz), *δ* = 21.3 (OCOCH_3_), 26.4 (C-13′), 28.9 (C-11′), 30.8 (C-12′), 31.6 (C-10′), 36.0 (C-1′), 45.00 (C-2′), 45.04 (C-4′), 67.4 (C-3′), 72.0 (C-5′), 100.9 (C-8′), 118.5 (C-6′), 170.4 (OCOCH_3_), 198.0 (C-9′), 209.5 (C-7′); ESI-Orbitrap-MS, calcd. for C_15_H_23_O_4_^+^ [M + H]^+^: 267.1591, found *m*/*z*: 267.1595.

### 3.3. Antiproliferation Activity of Caco-2 Cells by apo-13-Fucoxanthinone (***2***) and apo-9′-Fucoxanthinone (***3***)

Caco-2 cells were cultured in DMEM supplemented with 0.1 mM nonessential amino acids, 10% heat-inactivated fetal bovine serum, 4 mM L-glutamine, and antibiotics (40 units/mL penicillin and 40 mg/mL streptomycin) [36]. The culture was carried out at 37 °C in a humidified atmosphere with 5% CO_2_ in air. In order to evaluate the effects of these compounds on the viability of the cells, the cells were seeded at a density of 5 × 10^3^ cells per well containing 100 μL of culture medium in 96-well plates for 24 h, and fresh medium was used for the treatment with the compounds described below. Media containing the compounds were prepared using a liquid-drying method, as in our previous study [37]. In brief, fucoxanthin and the degraded compounds dissolved in the purified tetrahydrofuran (THF) and were added to the culture medium. The control culture received only THF in the medium (vehicle alone). The THF in the medium was dried in a centrifugal evaporator. The medium was passed through a 0.2-μm filter to be sterilized, and was then used as fresh medium supplemented with the compounds. To determine the concentration of the compounds, one part of the fresh medium was diluted 41-fold with ethanol for apo-13-fucoxanthinone (**2**) and apo-9′-fucoxanthinone (**3**), or with dimethyl sulfoxide/methanol (2:7, *v*/*v*) for fucoxanthin (**1**), respectively, and subjected to HPLC analysis. After 72 h of cultivation, cell viability was evaluated by 3-(4,5-dimethylthiazol-2-yl)-2,5-diphenyl tetrazolium bromide (MTT) assay [31]. Data represent means ± standard deviations. The antiproliferation experiments were done under dim yellow light in order to minimize the isomerization and degradation of xanthophylls due to light irradiation. The results were analyzed by one-way ANOVA and the Tukey–Kramer test in order to identify significant differences between treatments, with *p*-values < 0.05 considered significant.

## 4. Conclusions

We succeeded in decomposing fucoxanthin under very mild and neutral conditions. The decomposition obtained here had the same structure as degraded fucoxanthin found in nature. By refining HPLC more precisely, there is a good possibility of obtaining a new degradation product. Currently, more detailed isolation and purification processes are being conducted. In addition, it was found that the two types of degradation product obtained here cause growth suppression in Caco-2 cells. In particular, the compound with an allene structure preferentially inhibited proliferation compared to that without an allene structure. From this result, we predict that an allene structure is important to inhibit the proliferation of Caco-2 cells.

## Figures and Tables

**Table 1:** ^1^H-NMR and ^13^C-NMR chemical shift assignments (*δ* = ppm, CDCl_3_, reference TMS = 0.00 ppm) for peak A in Figure 2, apo-13-fucoxanthinone (**2**).

Comp. 2	^1^H	^13^C
C-1	-	35.1
CH_2_-2	1.34, 1.50	47.2
CH-3	3.81	64.2
CH_2_-4	1.79, 2.31	41.6
C-5	-	66.1
C-6	-	66.7
CH_2_-7	2.61, 3.63	41.4
C-8	-	198.1
C-9	-	143.1
CH-10	7.05	134.5
CH-11	7.47	136.7
CH-12	6.46	135.6
C-13	-	197.7
CH_3_-14	1.03	24.9
CH_3_-15	0.94	28.1
CH_3_-16	1.21	21.0
CH_3_-17	2.03	12.5
CH_3_-18	2.35	28.2

**Table 2:** ^1^H-NMR and ^13^C-NMR chemical shift assignments (*δ* = ppm, CDCl_3_, reference TMS = 0.00 ppm) for peak B in Figure 2, apo-9′-fucoxanthinone (**3**).

Comp. 3	^1^H	^13^C
C-1′	-	36.0
CH_2_-2′	1.44, 2.02	45.00
CH-3′	5.39	67.4
CH_2_-4′	1.53, 2.33	45.04
C-5′	-	72.0
C-6′	-	118.5
C-7′	-	209.5
CH-8′	5.87	100.9
C-9′	-	198.0
CH_3_-10′	1.16	31.6
CH_3_-11′	1.43	28.9
CH_3_-12′	1.43	30.8
CH_3_-13′	2.19	26.4
CH_3_-Ac	2.05	21.3
C-Ac	-	170.4

## Supplementary Materials

The following are available online at http://www.mdpi.com/1660-3397/16/8/275/s1, Figure S1: HSQC-NMR chart of apo-13-fucoxanthinone (**2**), Figure S2: HSQC-NMR chart of apo-9′-fucoxanthinone (**3**).
