# Supplementary material for: Degradation of Fucoxanthin to Elucidate the Relationship between the Fucoxanthin Molecular Structure and Its Antiproliferative Effect on Caco-2 Cells
Source: Mar Drugs. 2018 Aug 6;16(8):275. doi: 10.3390/md16080275 (PMC6117710; doi:10.3390/md16080275)
Supplement: Supplementary file 1 [file marinedrugs-16-00275-s001.pdf]

Supplementary Materials

Degradation of Fucoxanthin to Elucidate the Relationship between the Fucoxanthin Molecular Structure and Its Antiproliferative Effect on Caco-2 Cells

Shiro Komba *, Eiichi Kotake-Nara and Wakako Tsuzuki

Food Component Analysis Unit, Food Research Institute, National Agriculture and Food Research Organization, 2-1-12, Kannondai, Tsukuba, Ibaraki 305-8642, Japan; ekotake@affrc.go.jp (E.K.-N.); wakako@affrc.go.jp (W.T.)
*Correspondence: skomba@affrc.go.jp; Tel.: +81-29-838-7298

HSQC-NMR of compound **2** S2

HSQC-NMR of compound **3** S3

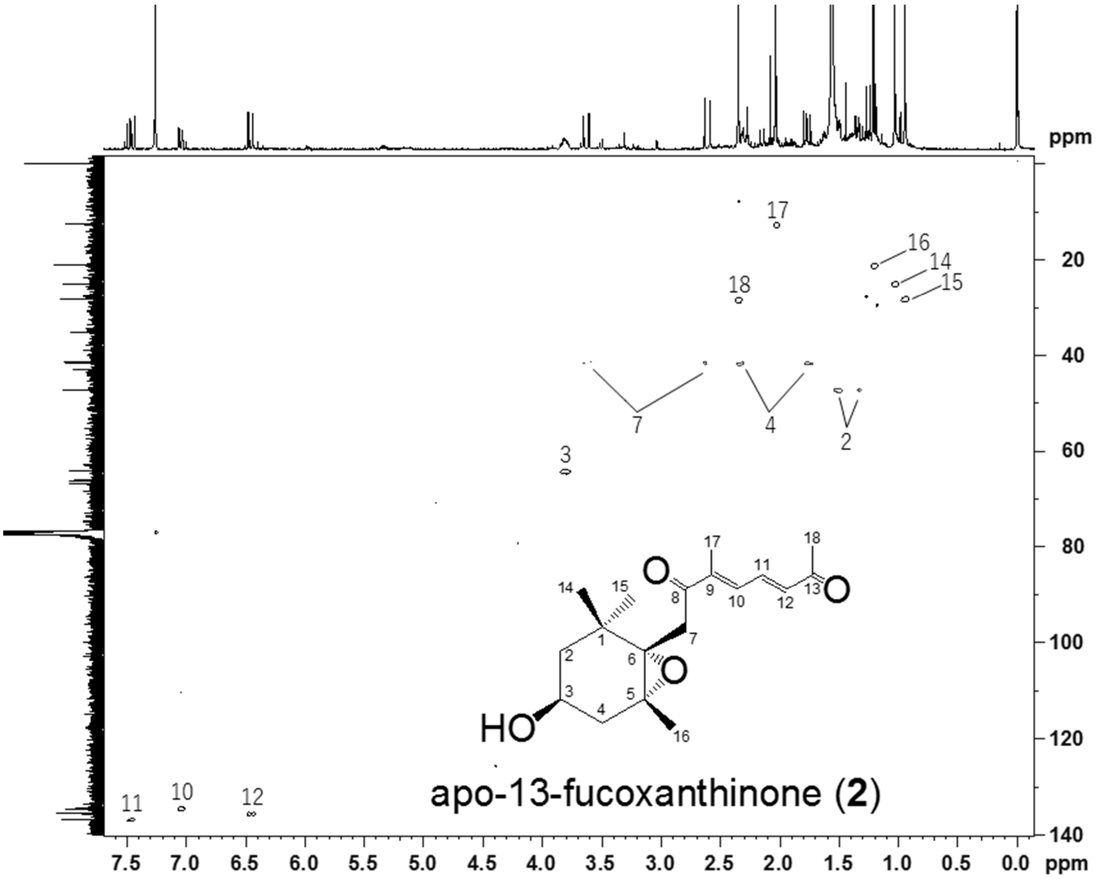

Figure S1. HSQC-NMR of apo-13-fucoxanthinone (2)

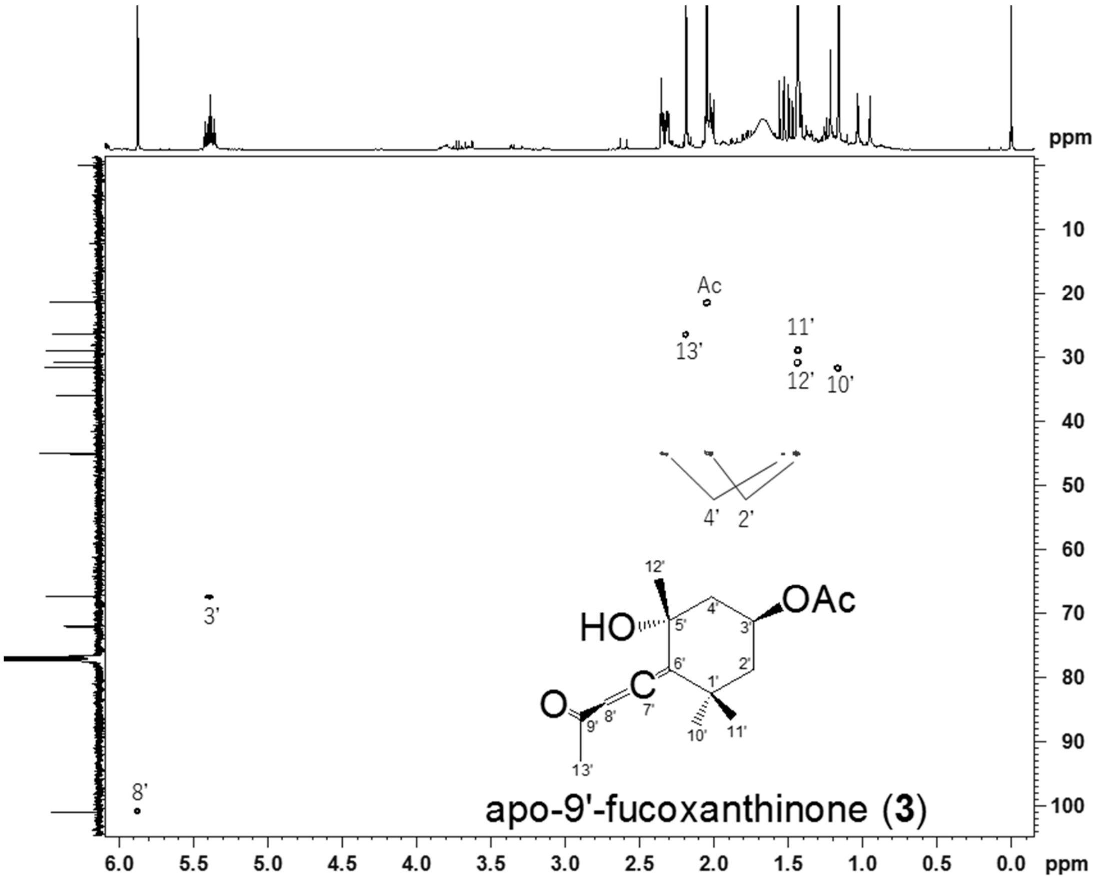

Figure S2. HSQC-NMR chart of apo-9'-fucoxanthinone (3)
